# Visible Light Is a Better Co-Inducer of Apoptosis for Curcumin-Treated Human Melanoma Cells than UVA

**DOI:** 10.1371/journal.pone.0079748

**Published:** 2013-11-08

**Authors:** Stephan Buss, Jadranka Dobra, Kerstin Goerg, Stephanie Hoffmann, Stefan Kippenberger, Roland Kaufmann, Matthias Hofmann, August Bernd

**Affiliations:** Department of Dermatology, Venereology, and Allergology, University Hospital, J.W. Goethe-University, Frankfurt/Main, Germany; University of Tennessee, United States of America

## Abstract

Curcumin attracts worldwide scientific interest due to its anti-proliferative and apoptosis inducing effects on different tumor cells at concentrations ranging from 10 to 150 µM (3.7–55 µg/ml). Unfortunately, because of a low oral bioavailability, only low and pharmacologically ineffective serum levels are achievable. In this study, an alternative treatment concept consisting of low concentration curcumin (0.2–5 µg/ml) and irradiation with UVA or visible light (VL) has been tested. The experimental results show clearly that this treatment decreases the proliferation and the viability of human melanoma cells while the cell membrane integrity remains intact. We identified the onset of apoptosis characterized by typical markers such as active caspases 8, 9 and 3 as well as DNA fragmentation accompanied by the loss of cell adhesion. The mitochondrial apoptosis signaling pathway is predominant due to an early activation of caspase-9. The present data indicate a higher efficacy of a combination of curcumin and VL than curcumin and UVA. Reduced effects as a result of light absorption by heavily pigmented skin are unlikely if VL is used. These results indicate that a combination of curcumin and light irradiation may be a useful additional therapy in the treatment of malignant disease.

## Introduction

Curcumin is an intensive orange-yellow colored, phenolic pigment found in the rhizome of the *Curcuma longa* plant. In the form of yellow powder, curcuma longa has been widely used as a spice and medicinal plant in traditional Asian medicine [1], [2]. Curcumin has attracted worldwide scientific interest regarding its anti-inflammatory, anti-oxidative and, as frequently demonstrated, anti-proliferative, and apoptosis-inducing effects on different tumor cells [2]–[4]. Concerning pigment producing cells and tumor cells, the influence of curcumin on pigmentation may be of special interest. Pigment synthesis is a complex regulated process involving many different factors acting in both a receptor-dependent and -independent manner via different signaling pathways. The complex mechanism of melanogenesis and its regulation has been extensively reviewed by Slominski et al. [5] Curcumin is known to inhibit melanin synthesis by down-regulation of microphthalmia associated transcription factor (MITF) and its downstream signal pathway through activation of PI3/AKT and the MAP-kinases ERK or p38 [6], [7]. In particular, the minimal side-effects of curcumin even at high oral administered doses of up to 8 g per day make it an interesting compound for treating malignant diseases [3]. Unfortunately, the effective usage of curcumin is hampered by its extensive metabolism by cytochromes P-450 (CYPs), UDP-glucuronosyltransferase (UGT), and sulfotransferase (SULT) [8]. Even a high-dose oral administration of curcumin leads only to low serum levels of<2 µM (<0,7 µg/ml) for a few hours [3] offering no significant pharmacological effects [9]–[12]. In this context, the discovery of a phototoxic activity of curcumin by Tonnesen et al. [13] and Dahl et al. [14], [15] is of particular interest as low concentrations are shown to be effective in achieving anti-proliferative and cytotoxic effects on bacteria and mammalian cells.

The structure of curcumin contains several functional groups which are pivotal for its biologic activity comprising two phenol groups, each with monosubstituated methoxy and hydroxyl groups, which are bound together by an aliphatic carbohydrate chain with a diketone group [1], [16]. The keto groups of the molecule exhibit potent electrophilic characteristics [2] but also the methoxy groups of curcumin are important for its anti-oxidative properties [1]. A central role for the apoptosis inducing effect of curcumin is played by the electrophilic double bonds of the carbohydrate chain that can be attacked by a nucleophilic group in an addition-reaction forming a covalent adduct [17], [18]. Furthermore, the lipophilic curcumin interacts with hydrophobic protein domains, dissolves in cellular lipid bilayers and disturbs the fluidity leading to a change in the conformation and function of integral membrane proteins [2], [19], [20]. Overall, it can be assumed that because of these multiple biochemical properties of curcumin, a major molecular mechanism could not be identified so far [2].

Despite the still unknown exact biochemical mode of action of curcumin, there are many publications available describing its effects on cellular signaling cascades. The first step is a cell cycle arrest at the G2/M transition point, but also at transition points G0/G1- and G1/S, resulting in inhibition of cell proliferation [21]–[24]. An increase in curcumin dose (>10 µM) and incubation time (24 h) ultimately cause apoptosis in several tumor cells [4], [9], [11], [25]–[30]. Most studies describe the curcumin-induced apoptosis as caspase-dependent with a documented activation of effector caspase-3 [4], [9], [11], [25]–[29], [31]–[32]. Several researchers came to different conclusions regarding the role of the apoptotic pathways: some suggest the mitochondrial pathway [4], [28], [32], some the extrinsic pathway via caspase-8 [34], [35] and others both pathways as being predominant [10], [36], [37]. There are also some studies suggesting a predominant role of caspase-independent signaling pathways in curcumin-induced apoptosis [38], [39]. It seems that the induction of apoptosis by curcumin is dependent on the cell line used and on the interaction of curcumin with several proliferation associated factors. Curcumin inhibits the kinases PKA and PKC, the receptor tyrosine kinase EGFR and the transcription factors NFκB and c-myc (summarized in [3], [26]). Taken together, this leads to a down-regulation of anti-apoptotic signaling molecules such as Bcl-2, Bcl-X_L_, cIAP, survivin, TRAF1 and cyclin D1 in downstream pathways [25], [32]. The impairment of many apoptotic factors based on the multiple biochemical properties of curcumin may be responsible for apoptosis induction in variably differentiated cells [2], [3], [25].

Based on the observation that low concentrations of curcumin combined with light irradiation (UVA and VL) induce apoptosis in human keratinocytes [12], we tested the effects of such a combined treatment on human melanoma cells.

## Materials and Methods

### Cell culture

In this study, melanoma cancer cell lines G-361 and A375 were used. G-361, and A375 tumor cells were obtained from the American Type Culture Collection. G-361 cells were grown in RPMI 1640 medium (Gibco, Karlsruhe, Germany) with 10% FBS, 1% P/S solution and additional 1% L-glutamine solution (Invitrogen, Karlsruhe, Germany). The A375 cell culture medium contained Dulbecco Modified Eagle Medium with 4.5 g/l D-Glucose (DMEMhigh) (Gibco, Karlsruhe, Germany), 10% FBS and 1% P/S solution. All cells were cultured at 37°C in a humidified atmosphere containing 5% CO_2_ in case of G-361 cells and 7.5% CO_2_ in case of A375 cells.

### Treatment

All experiments were performed under sterile conditions; all media used were warmed to 37°C. HEPES buffer was added to the culture media needed for experiments (40 mM), the pH values remained constant at 6.8. A curcumin stock solution was prepared by dissolving 30 mg of curcumin (Sigma-Aldrich, Taufkirchen, Germany) in 1 ml DMSO. Serial dilutions of curcumin were prepared from the stock solution using culture medium or phosphate buffered saline (PBS). The highest final concentration of DMSO was 0.02% which is about 50 fold lower than the described toxic concentrations [40] and did not show any detectable effect on cell behavior (not shown). Cells were detached from the culture flasks using trypsin/EDTA and seeded into the required test vessel. After 24 hours, the adherent cells were incubated with curcumin-containing medium for 1 hour. Prior to irradiation the medium was replaced by PBS to prevent a build-up of reactive photochemical products. Cells were then irradiated with UVA (315–400 nm, cumulative dose 1 J/cm^2^, Waldmann, Villingen-Schwenningen, Germany) or visible light (5500 lx, 10×40 W lamps, distance 45 cm, emission spectrum: 400–550 nm, cumulative dose 1.65 J/cm^2^; Philips GmbH, Hamburg, Germany). Under the described conditions the visible light source emitted a small UVA part with a cumulative dose of 0.03 J/cm^2^ measured with an UV-meter (Waldmann, Villingen-Schwenningen, Germany). A rise in temperature was excluded by continuous measurements with a mercury thermometer. Parallel to the irradiation, identically treated controls were kept in the dark. Thereafter, culture medium was added and the test vessel kept in an incubator until the analysis 4–48 hours later. In some experiments synthetic melanin was added to the culture medium to check whether this pigment lowers the light intensity by absorption. For this purpose a stock solution of melanin was prepared by dissolving 500 µg/ml melanin (Sigma, Taufkirchen, Germany) in PBS at 95°C for 6 hrs. After cooling to 37°C the used concentrations were generated by diluting the stock solution with culture medium.

### Cell proliferation, viability and cytotoxicity

The proliferation rate was measured using the cell proliferation BrdU ELISA Kit (Roche Diagnostics, Penzberg, Germany) and quantification of cell viability was conducted by the Alamar Blue test (AbD Serotec, Oxford, UK). To determine the toxicity of the treatment leading to cell lysis the cytotoxicity detection kit (Roche Diagnostics, Mannheim, Germany) was used. Cells were seeded in 96-well microtiter plates with a density of approximately 1×10^4^ cells/well for viability and proliferation tests and 1.5×10^4^ cells/well for cytotoxicity tests. 24 hours later cells were treated as described; the range of curcumin concentrations used was 0.2 to 2 µg/ml (0.68–5.43 µM).

#### Cell proliferation BrdU ELISA

To determine the DNA replication rate, 5-bromo-20-deoxyuridine (BrdU) was added to the cells immediately after irradiation. 24 hours later the cells were fixed and peroxidase-coupled BrdU-antibodies were added to form BrdU-bound immune complexes. Next, the peroxidase substrate tetramethylbenzidine was added; the resulting color change was measured using an ELISA reader (Expert 96, ASYS, Eugendorf, Germany; 450 nm).

#### Alamar Blue test

Analysis started after the final incubation period of 24 hours by replacing the medium with PBS and addition of Alamar Blue reagent (10%). The reduction of the blue dye resazurin to the red fluorescent resorufin - as an equivalent to the mitochondrial metabolic activity - was measured after 2 hours using a CytoFluor fluorescence reader (series 4000, PerSeptive Biosystems, Framingham, MA; excitation 530 nm, emission 620 nm).

#### Cytotoxicity detection

Cells of the positive control were treated with 1% Triton-x-100. All other cells were treated as described above. After the final incubation period of 24 hours, cell-free supernatants containing lactate dehydrogenase from lysed cells were obtained. The added NAD^+^ solution was reduced during the lactate dehydrogenase reaction to NADH/H^+^, which changed the yellow tetrazolium salt to a red-colored formazan salt. The resulting color change was measured using an ELISA reader (Expert 96, ASYS; 490 nm).

### Apoptosis

Central characteristics of apoptosis are the DNA fragmentation into 180 base pair long multimeres and the caspase activation. To quantify the fragmentation rate the Cell Death Detection ELISA (CDD) (Roche, Mannheim, Germany) was used. Caspase activation was documented by the Western blot technique. For CDD, cells were seeded in 96-well microtiter plates with a density of approximately 1×10^4^ cells/well. For western blot analysis cells were seeded in three-well multidishes (1.25×10^6^ cells/well). 4–12 hours later cells were treated as described. The curcumin concentrations used ranged from 0.2 to 5 µg/ml (0.68–13.57 µM).

#### Cell Death Detection ELISA

After the combined treatment and 15 hours of incubation, culture medium was discarded and the adherent cells were lysed. The lysate containing DNA fragments was transferred on to streptavidine coated strips. Next, an antibody enriched solution (anti-histone-biotin-antibody, anti-DNA-peroxidase-antibody) was added leading to stable sandwich-like immune complexes. The enzymatic conversion of the peroxidase substrate azino-ethylbenzothiazoline-sulfonic-acid to a colored product was measured using an ELISA reader (Expert 96, ASYS; 405 nm).

#### Western blot

All cells were lysed with 3×SDS sample buffer (187.5 mM Tris–HCl at pH 6.8, 6% SDS, 30% glycerol, 150 mM dithiothreitol, 0.3% bromphenol blue) 4 hours after the combined treatment. The lysates were collected, sonicated and boiled for 5 minutes at 95°C. Then the samples were electrophoresed on 15% SDS-PAGE gels in Laemmli-buffer (25 mM Tris, 250 mM glycine, 0.5% SDS) at 120 V. Next, the proteins were transferred to a polyvinylidene fluoride membrane (PVDF, Millipore, Bedford, MA) using towbin buffer (25 mM Tris, 192 mM glycine, 10% methanol) at 50 V. Incubating for 30 min in blocking buffer (Tris-buffered saline (pH 7.6), 0.1% Tween-20, and 5% BSA or non-fat dry milk) blocked non-specific binding sites. Afterwards, PVDF-membranes were covered with specific primary antibody solutions (blocking buffer) against caspase-9 (1∶3000), caspase-8 (1∶3000), caspase-3 (1∶3000, Cell Signaling, Beverly, USA) and beta-actin (1∶5000, Sigma-Aldrich, Taufkirchen, Germany) antigens for 24 hours. Detection of the primary antibodies was carried out using secondary, horseradish peroxidase-linked antibodies (Cell Signaling). Finally, the antigen–antibody complexes were visualized by a chemiluminescence detection system (LumiGlo solution, Cell Signaling; Hyperfilm-ECL, Amersham Pharm., Little Chalfont, UK; PROTEC C35, Protec, Oberstenfeld, Germany).

### Statistics

For each curcumin concentration to be tested, cells were seeded in 8 wells (cell viability, proliferation and cytotoxicity), 4 wells (CDD detection) or 1 well (western blot). Each combination possibility was tested separately (no irradiation, irradiation with UVA, irradiation with visible light). Mean value and standard deviation were calculated from 8 or 4 single values using the program Microsoft excel. All mean values as well as the standard deviation were expressed as a percentage of the completely untreated control. The statistical difference of the values referred to the untreated control was determined by the Mann-Whitney U-test. A *P*-value of ≤0.05 was considered significant. All experiments were repeated at least twice.

## Results and Discussion

### Impairment of DNA synthesis and viability by curcumin/light

**Figure 1:**
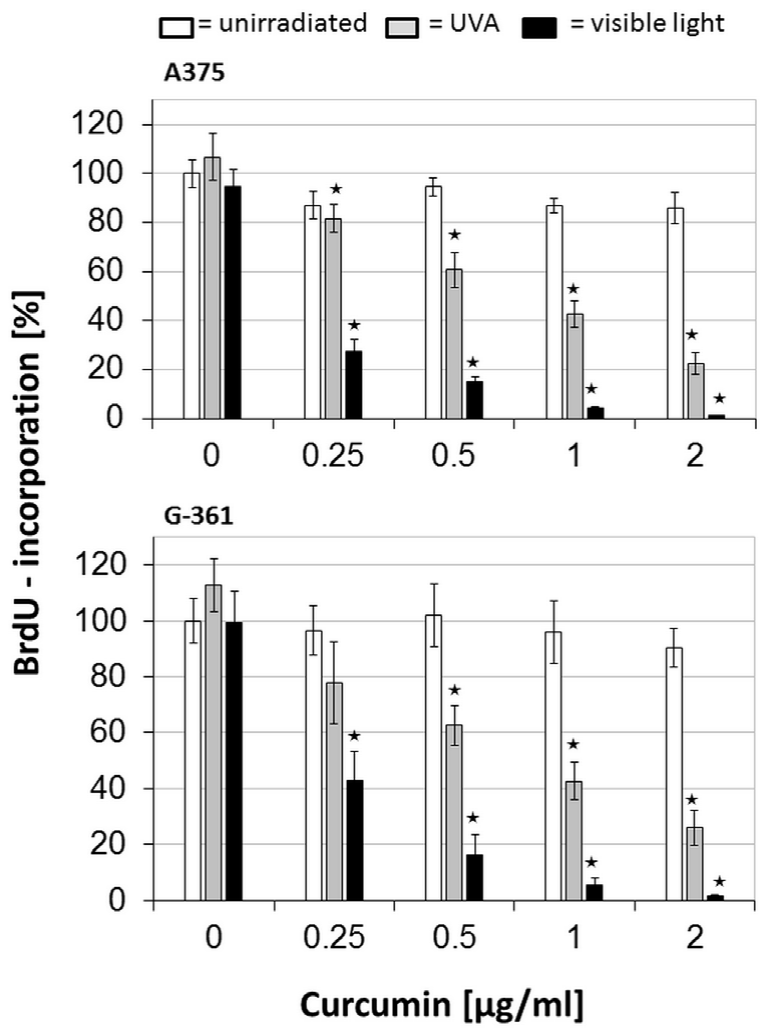
DNA synthesis in melanoma cells (A375 and G-361) after treatment with curcumin and light: After incubation with curcumin, cells were irradiated with UVA (gray bars), or visible light (black bars), or remained light-protected (white bars). All values were referred to the untreated control as a 100% reference. Every bar represents the average of 8 simultaneously determined individual values; standard deviations are shown at the top. Highly significant reduction of DNA synthesis of the treated cells is marked with * (*P*≦0.001).

**Figure 2:**
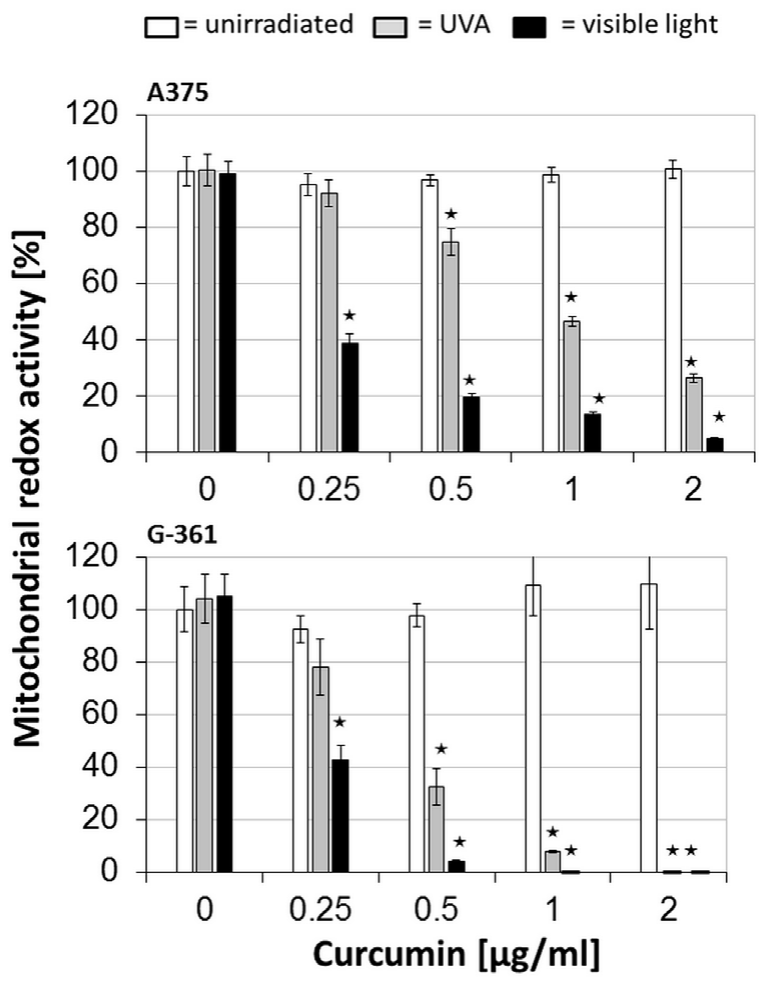
Mitochondrial redox activity in melanoma cells (A375 and G-361) after treatment with curcumin and light: After incubation with curcumin, cells were irradiated with UVA (gray bars), or visible light (black bars), or remained light-protected (white bars). All values were referred to the untreated control as a 100% reference. Every bar represents an average of 8 simultaneously determined individual values; standard deviations are shown at the top. Highly significant reduction of viability of the treated cells is marked with * (*P*≦0.001).

The absorption of light by melanin reaches its maximum at a medium UVA range (≈350–330 nm) [41], but a second absorption maximum of curcumin is within the visible wavelength range (420 nm) [13], therefore, a better response to the treatment curcumin/visible light (VL) is proposed. The results of the BrdU-based proliferation tests showed that the combined treatment inhibits the proliferation of melanoma cells in a concentration dependent manner. As shown in figure 1, the first inhibitory effects were observed at a curcumin concentration of 0.2 µg/ml combined with UVA or VL irradiation. The combination curcumin/VL was more effective giving an inhibition rate of nearly 95–98% at 1 µg/ml whereas curcumin/UVA caused a maximum inhibition of 75–95% at 2 µg/ml. In the controls, neither curcumin alone nor UVA or VL irradiation alone induced a highly significant inhibition of DNA synthesis. These results prove not only a photo-activation of curcumin but also that a photo-activation is necessary when using low concentrations. These findings agree with the observations of Dujic et al. [12], [42] regarding a better effect of curcumin/VL. A few recently published studies on the effect of low curcumin doses combined with light irradiation confirm these results as reviewed by Bernd [43]. Data from experiments investigating the cell viability confirmed these results (figure 2). Initial inhibitory effects started again at a curcumin concentration of 0.2 µg/ml. We detected a concentration-dependent impairment of mitochondrial activity and a greater effect of curcumin and VL compared to curcumin and UVA similar to previous experiments regarding DNA synthesis. Curcumin or irradiation alone had no effect. Investigating the effects of 3.68 µg/ml (10 µM) curcumin on cellular signaling cascades, studies by Park et al. [21] and Liu et al. [22] showed the induction of cell cycle arrest at the G2/M transition point in urinary bladder carcinoma cells and in glioma cells. In our experiments, curcumin concentrations of 2 µg/ml (5.43 µM) had no effect on DNA synthesis (Fig. 1 and 2). The impairment of DNA synthesis and mitochondrial redox activity by the combined treatment implies that cells were more likely forced into cell death than into a possible cell cycle arrest.

### Role of melanin in a possible clinical application

**Figure 3:**
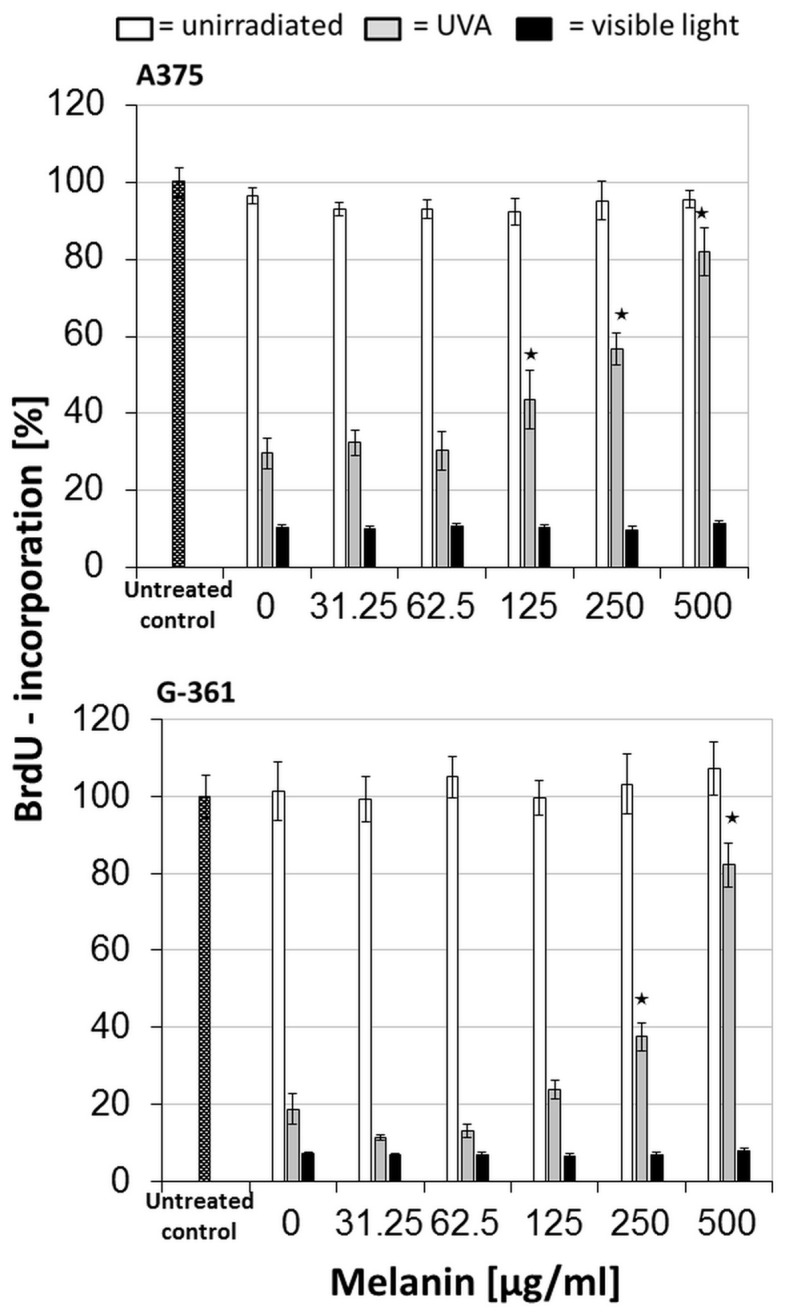
Influence of melanin on the treatment with curcumin/light: After incubation with 2 µg/ml curcumin, a curcumin containing PBS with increasing melanin concentrations was added. Cells were irradiated with UVA (gray bars), or visible light (black bars), or were light-protected (white bars) followed by the determination of the BrdU-incorporation rate. All values were referred to the untreated control (dotted bars) as a 100% reference. Every bar represents the average of 8 simultaneously determined individual values; standard deviations are shown at the top. Compared to the treated controls (0 µg/ml melanin), highly significant reduction of proliferation inhibition is marked with * (*P*≦0.001). Upper Part: A375; lower part: G-361 melanoma cells.

Although the investigation of proliferation and viability of treated cells provided no hint of an absorptive effect of melanin in vitro, this question remained taking into account the multilayered architecture of human skin. Using the measurements of other groups correlating skin pigmentation and melanin concentration, melanocytes used in other studies could be assigned to the highly pigmented skin photo-types IV - VI [44). Due to supplying approximately 40 supra-basal keratinocytes with melanin provided by one melanocyte and because of aggregation of melanin to nuclear caps in the path of light, cumulative effects in human skin are to be expected [44], [45]. To simulate a multilayered pigmentation, in addition to 2 µg/ml curcumin, synthetic melanin was added to the medium in increasing concentrations. After irradiation with UVA or VL, cell proliferation was determined as described previously. The results of these trials, presented in figure 3, demonstrate a significant mitigation of the efficacy of curcumin and UVA, when 125 µg/ml or even more melanin was added. In contrast, treatment with curcumin and VL was not affected even by high melanin concentrations, causing a maximum inhibition of proliferation. Un-irradiated controls which either were treated with curcumin alone or with curcumin and melanin, were not impaired. According to the observation that additionally added melanin reduces the effectivity of the treatment combined with UVA, absorptive disturbances can be expected in heavily pigmented skin. This is another reason to prefer the combination curcumin and VL in possible clinical applications.

### Membrane integrity remained unaffected

**Figure 4:**
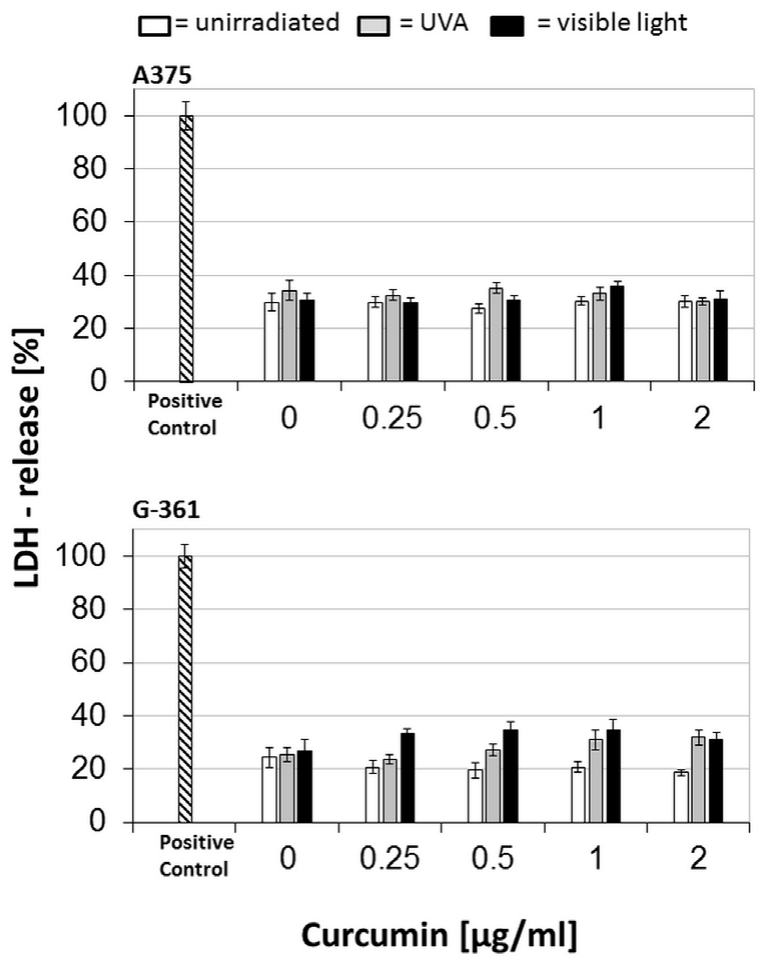
LDH release in melanoma cells (A375 and G-361) after treatment with curcumin and light: After incubation with curcumin, cells were irradiated with UVA (gray bars), or visible light (black bars), or were light-protected (white bars). Cells of the positive control were treated with 1% triton-X100 containing medium (dashed bars). All values were referred to the positive control as a 100% reference. Every bar represents the average of 8 simultaneously determined individual values; standard deviations are shown at the top.

Hallmarks of necrotic cell death are loss of cell membrane integrity and release of pro-inflammatory cytosolic components triggering inflammation in vivo [46]. To exclude a necrotic cell death, which is undesirable in clinical use, we investigated the levels of the cytosolic enzyme lactate dehydrogenase (LDH) in culture medium of treated cells. The results displayed in figure 4 show that no curcumin concentration dependent release of LDH into the medium occurred. Also, the UVA and VL irradiation alone did not increase the LDH levels compared to untreated controls. In the case of G-361 cells, slightly increased LDH levels were measured, mainly affecting those cells treated with curcumin/VL. However, the extent of LDH release did not compare with the induced decrease of DNA synthesis and mitochondrial activity. Therefore, membrane damage as the underlying mechanism after the treatment with curcumin/light seems unlikely. These experiments were also interpreted against the background of several experimental results showing conflicting properties of curcumin on cellular levels of reactive oxygen species (ROS). Rising intracellular concentrations of ROS lead to oxidative damage of DNA, loss of enzymatic activity of proteins and, via lipid peroxidation, to a change in membrane fluidity. Thus, ROS can culminate in proliferation inhibition, in apoptotic cell death, and in necrotic cell death [47], [48]. Increased ROS levels were detected in ovarian cancer cells and in human osteoblasts when using 10–100 µM curcumin causing cell cycle arrest, apoptosis, and necrosis [31], [33]. On the other hand, Fiorillo et al. [49] and Zhu et al. [16] showed an anti-oxidative effect of 2.5–20 µM curcumin on human cardiac cells and on cortical neurons. Since Dujic et al. [12] demonstrated an anti-oxidative effect of 0.2–1 µg/ml (0.68 – 2.71 µM) curcumin which neutralized ROS generated by high dose UVA-irradiation, a dose dependent dual role of curcumin seems likely with a turning point at 3.68–7.36 µg/ml (10–20 µM). Therefore, a change in intracellular ROS in our treatment protocol is presumably not the case.

### Induction of DNA fragmentation by curcumin/light

**Figure 5:**
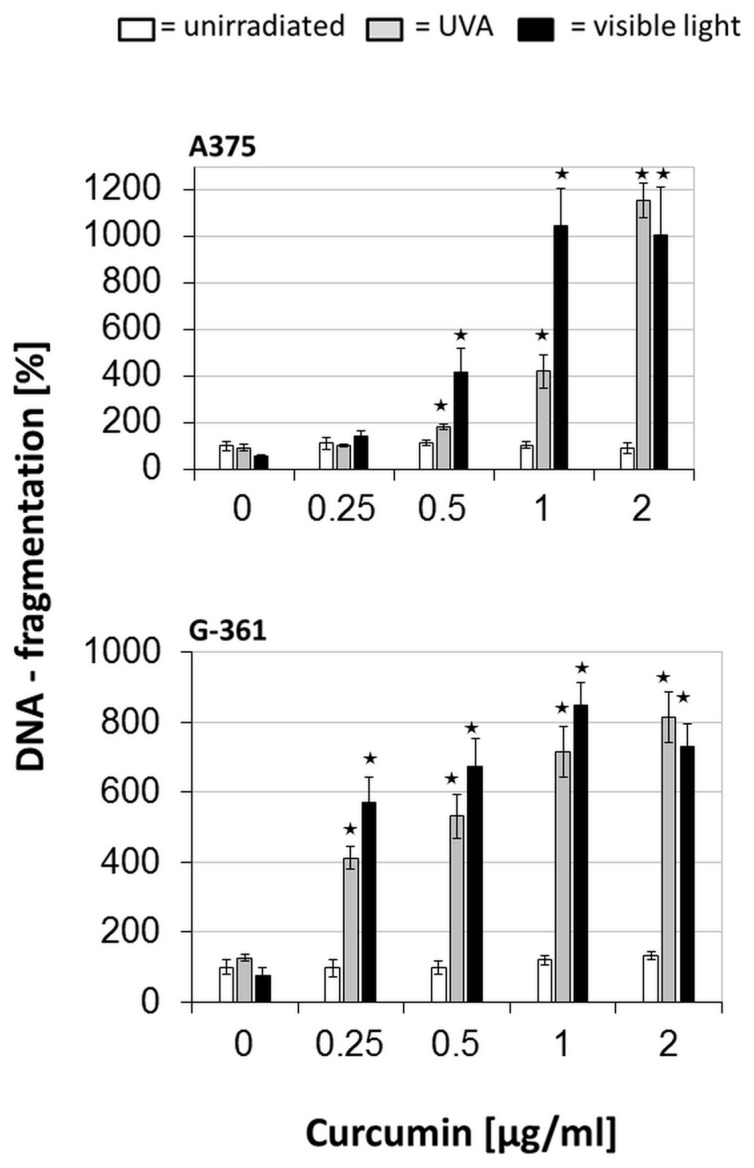
DNA fragmentation in melanoma cells (A375 and G-361) after treatment with curcumin and light: After incubation with curcumin, cells were irradiated with UVA (gray bars), or visible light (black bars), or were light-protected (white bars). All values were referred to the untreated control as a 100% reference. Every bar represents the average of 4 simultaneously determined individual values; standard deviations are shown at the top. Significant increase of DNA fragmentation of the treated cells is marked with * (*P*≦0.05).

According to our results and to observations of Dujic et al. [12] that curcumin/light induces apoptosis in human keratinocytes, an induction of apoptosis in treated melanoma cells was considered. Hence, typical characteristics of apoptosis such as DNA fragmentation and caspase activation [50] were investigated. Figure 5 demonstrates the analysis of the DNA-fragmentation rate in treated melanoma cells showing DNA fragmentation in treated cells in a concentration dependent manner. Using a combination with VL, DNA fragmentation started at a curcumin concentration of 0.2 µg/ml in melanoma cells. In the case of UVA irradiation, DNA fragments did not occur until a curcumin concentration of 0.5 µg/ml was reached (figure 5). Again these findings reflect a higher efficacy of curcumin and VL revealing a high susceptibility of melanoma cells towards the combined treatment. Signal fluctuations occurred because apoptotic cells increasingly detach from adjacent tissue [50]. Based on the procedure of the cell death detection ELISA (CDD-ELISA), cell culture medium was discarded prior to cell lysis leading to loss of apoptotic bodies. In melanoma cells, signal stagnation occurred between 10 and 24 hours. These results demonstrate a rapid onset of apoptosis and a loss of cell adhesion in combined treated cells after irradiation. Apoptosis induction proceeded more rapidly when a higher dose of curcumin and VL irradiation was used.

### Induction of caspase activation by curcumin/light

**Figure 6:**
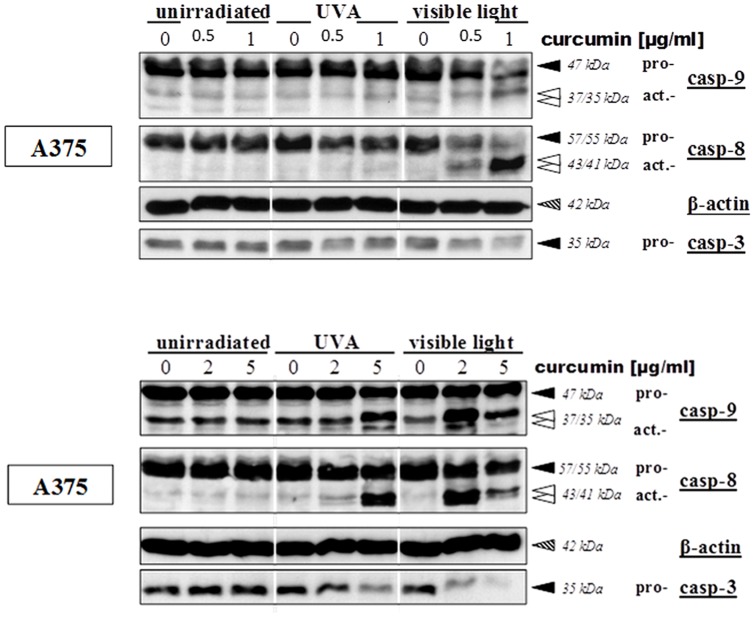
Caspase activation after treatment with curcumin (upper part: 0.5 and 1 µg/ml; lower part: 2 and 5 µg/ml) and light: A375 melanoma cells were incubated with curcumin and thereafter irradiated with UVA (middle lanes) or visible light (right lanes). Controls remained light-protected (left lanes). After 6 hours cells were lysed. Detection of inactive (black arrows) and active (white arrows) forms of caspase-8, caspase-9 and caspase-3 was carried out using the Western blot technique. β-actin-bands represent the loading control (dashed arrows).

Using the Western blot technique we studied caspase activity as an early marker of apoptosis. Initial experiments using curcumin concentrations of 0.5 and 1 µg/ml showed only an activation of caspases in VL-irradiated cells (figure 6). Thus, the subsequent tests included incubation with 2 and 5 µg/ml curcumin and cell lysis 4 hours after irradiation. The results presented in figure 6 show increasing signals of active caspases [11], [12] and declining signals of procaspase-3, an activation of initiator caspases-8 and -9, as well as activation of the effector caspase-3. Remarkably, caspase activation in UVA-irradiated cells was first noticeable at 2 µg/ml and reached a maximum at a curcumin concentration of 5 µg/ml (figure 6). An irradiation with VL induced a maximum signal at 2 µg/ml followed by a signal loss at 5 µg/ml - both corresponding to previous results indicating a higher efficacy of this treatment. Curcumin concentrations ranging from 0.5 to 5 µg/ml in combination with VL definitely induce apoptosis in melanoma cells. Several studies by others have highlighted the pro-apoptotic properties of curcumin on different cell types. In contrast to our findings in the present study, high concentrations of curcumin ranging from 10 to 50 µM combined with a long-term incubation of 8–72 hours had to be used [9], [10], [51]. Other studies demonstrated that prior to apoptosis cell cycle was arrested at specific transition points, particularly in the G2/M transition point, but also at transition points G0/G1- and G1/S [4], [26]–[29]. Our results suggest that apoptosis induction occurs in melanoma cells after treatment with low doses of curcumin combined with light irradiation.

### Intrinsic apoptotic pathway is predominant

**Figure 7:**
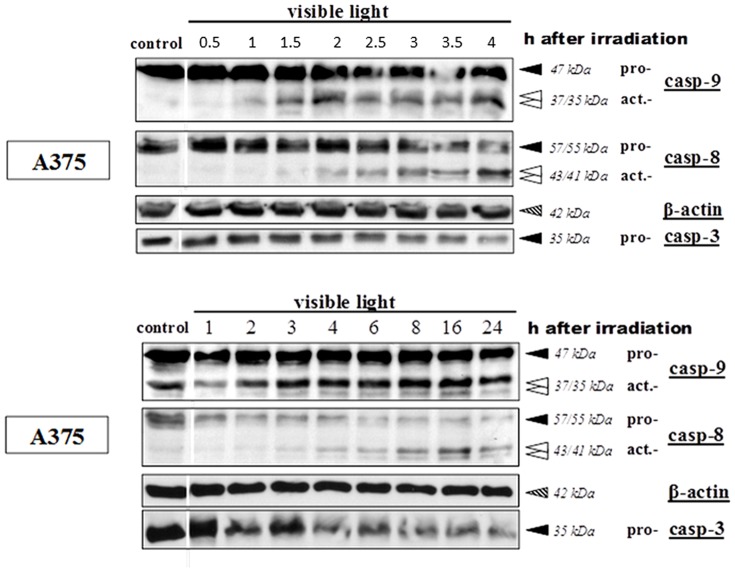
Chronological investigation of caspase activation after treatment with curcumin and light: A375 melanoma cells were incubated with 5 µg/ml curcumin and afterwards irradiated with visible light. Controls remained light-protected. After different periods of time (upper part: 0.5–4 hours; lower part: 1–24 hours) cells were lysed. Detection of inactive (black arrows) and active (white arrows) forms of caspase-8, caspase-9 and caspase-3 was carried out using the Western blot technique. β-actin-bands represent the loading control (dashed arrows).

Time-dependent lysis of cells displayed in figure 7, activated caspase-9 was detected already 1.5–2 hours after VL-irradiation. Contrary to some publications denoting caspase-8 [34], [35] or caspase-8 and -9 as leading initiator caspases in curcumin-induced apoptosis [10], [36], [37], curcumin/light clearly induced the intrinsic apoptosis pathway. Activation of caspase-8 became evident only 2 hours (and later) after irradiation which can be considered as a secondary effect in enhancing the apoptotic signaling cascade [52]. Similar observations were made by Dujic et al. [12] using keratinocytes. All these results also confirm the findings of Laubach et al. [53], which excluded the initiation of an extrinsic apoptosis via death receptors by the combined treatment with curcumin and light irradiation.

Due to the rapid apoptotic reaction kinetics in melanoma cells exposed to curcumin and light, the slight LDH release into the medium mainly after treatment with curcumin/VL (figure 3) has to be discussed. Since apoptotic cells in vivo increasingly detach from adjacent tissue prior to phagocytosis by neighboring cells and macrophages, in vitro apoptotic bodies undergo secondary necrosis after ATP depletion and failure of ion pumps [46], [50]. Kurbanov et al. [54] treated melanoma cells with TRAIL and measured, in addition to apoptotic hallmarks, an increased release of LDH after 24 hours, an effect not present 6 hours after treatment. Thus it can be concluded that most probably, secondary necrosis was present in some cases 24 hours after treatment with curcumin/light.

In this study we have shown clearly that curcumin concentrations of 0.25–5 µg/ml in combination with VL and of 0.5–5 µg/ml in combination with UVA induce apoptosis in treated melanoma cells. All results indicate a higher effectivity of the treatment combined with visible light, which induces apoptosis in up to 99% of all cells exposed to curcumin concentrations of 1–2 µg/ml (2.7–5.4 µM). Compared to curcumin alone, a treatment in combination with light irradiation offers several advantages regarding a possible clinical use. Since the required curcumin concentrations are low, oxidative effects can be excluded, which have been documented in several studies for high curcumin concentrations. Despite the extensive metabolism of curcumin, the low serum concentrations needed for the combined treatment can be achieved after oral application. When combined with light irradiation, the time- and dose-dependency of the mode of action of curcumin is amplified to a great extent and limited to the irradiated area only. Synergistic effects can be expected from an additional combination with cytostatic drugs. Overall, the results of this study indicate that the combined treatment of curcumin and light irradiation could be considered as an additional therapeutic concept of malignant diseases.
